# Definition of minimum clearance height of railway bridges in a windblown sand area based on numerical simulation

**DOI:** 10.1016/j.heliyon.2024.e41515

**Published:** 2024-12-26

**Authors:** Xian Zhang, Shengbo Xie, Yingjun Pang

**Affiliations:** a Research Laboratory of Desert and Desertification / Dunhuang Gobi and Desert Research Station, Northwest Institute of Eco–Environment and Resources, Chinese Academy of Sciences, Lanzhou, 730000, China; b University of Chinese Academy of Sciences, Beijing, 100049, China; c Institute of Ecological Conservation and Restoration, Chinese Academy of Forestry, Beijing, 100091, China

**Keywords:** Windblown sand area_1_, Wind-sand flow field_2_, Beam bottom clearance_3_, Railway bridge_4_, Numerical simulation_5_

## Abstract

Railway bridges with lower beam bottom clearances in windblown sand areas tend to accumulate sand particles on the sides of the beams, which seriously impacts railway safety. To investigate the effect of beam clearance height on wind-sand movement near the surface, and to determine the minimum clearance height for railway bridges in such areas, computational fluid dynamics using the Euler-Euler two-phase flow model was employed to simulate the wind-sand flow field beneath bridges with different heights. The results indicated that as clearance height increased, both the high-speed area above the bridge and acceleration area under the bridge increased, while the turbulence area on the leeward side remained unchanged. Furthermore, wind speed on the windward side did not decrease, and no deceleration zone near the surface was observed with increased clearance height. When the bridge height reached 9 m, the wind speed on the windward side no longer decreased. The correlation coefficients of near-surface wind speed under the bridge height of 9 m and above were consistent. As wind speed increased, the fluctuations in wind speed between bridges with different clearance heights became more pronounced, with differing variation trends. On the leeward side, lower clearance heights resulted in greater wind speed fluctuation. With higher wind speeds, the main position of wind speed deceleration moved further from the bridge. Therefore, the clearance height of bridges in the windblown area should be at least 9 m. These results provide a theoretical basis for the survey and design of bridges in the windblown sand areas.

## 1 Introduction

Windblown sand areas are characterized by surfaces dominated by sand and gravel, sparse vegetation, and a dry, arid environment. In these regions, wind-sand flow can pose significant threats to buildings and local transportation infrastructure [1]. With the development of arid and semi-arid regions and advancements in railway construction technology, more railways are being built in desert areas [2,3]. Consequently, the mileage of railways passing through sandy regions is increasing annually, compromising the safety of some routes [4]. Railway embankments, which are laid directly on the ground, obstruct the natural wind-sand flow, leading to issues such as erosion, burial, or abrasion [[5], [6], [7], [8]]. In contrast, bridges have a distinct structural advantage in mitigating the impact of wind-sand flow [9,10]. Bridges allow wind-sand flow to pass relatively smoothly beneath them, causing minimal changes to the surrounding environment. However, as the wind-sand flow passes over bridge, it disrupts the airflow, breaking both the energy balance and the wind-sand flow field beneath the bridge beam. The clearance height plays a crucial role in sand transportation. A lower clearance height slows down the wind speeds on either side of the bridge beam, leading to sand accumulation in these areas [11]. If the sand deposition around the bridge exceeds the clearance height, sand particles can jump onto the bridge platform and accumulate on the tracks. This phenomenon is particularly evident at the Basuoqu Bridge on the Qinghai-Tibet Railway, where the low clearance height causes frequent sand damage [12]. Sand deposition near the bridge's beam significantly affects railway safety [13]. Existing research suggests that higher bridges have less impact on near-surface wind-sand movement. If the clearance height is sufficiently high, the bridge does not affect the sand movement [14]. A proper clearance allows the wind-sand flow to pass smoothly, avoiding sand accumulation under the bridge. Therefore, determining the minimum clearance height for bridges in the wind-sand area is crucial.

Current research on railway sand hazards primarily focuses on sand flow structures [[15], [16], [17]], blown sand environments [18], the mechanism of sand damage formation [19], sand control systems, and their benefits [[20], [21], [22]]. Fewer studies have addressed bridge sand disasters, such as the causes of sand accumulation under bridges [13,23,24], the impact of sand movement on railway bridges [9,[25], [26], [27]], flow field structure of bridges [26], and sand control measures [27,28]. Some research has explored how sand accumulates beneath bridge beams, finding that clearance height plays a key role [13,25]. While it is known that windblown sand obstructed by bridges can cause sand hazards and that bridge clearance height affects sand accumulation. However, no systematic study has been conducted on how different clearance heights influence the flow field under bridges. There is no clear definition of the appropriate clearance height for sandy area.

The clearance height of railway bridges affects the sand movement beneath them, potentially leading to the sand particles deposition and compromising railway safety. Existing research primarily focuses on wind-sand flow field structures and the causes of sand damage. There is no study defining the minimum railway bridge clearance for railway bridges. This study uses numerical simulations to investigate how different clearance heights impact wind speed variations and wind flow field distribution under railway bridges in windblown sand area. Defining the minimum clearance height of railway bridges will help reduce sand hazards and provide a theoretical basis for the survey and design of railway bridges in these regions.

## 2 Materials and methods

### 2.1 Theoretical equation

In this research, the Euler-Euler two-phase model was used to study the wind-sand movement near the surface of the bridge. The model suggests the fluid-solid two-phase materials in the calculation domain as a continuous medium that penetrates each other. The sum of the two-phase volume fractions is 1, as shown in Equation (1). The gas phase and sand phase are controlled by their respective mass and momentum conservation equations, and there is an interaction force.

(1) $$\alpha_{g}+\alpha_{s}=1\text{.}$$

where *α*_*g*_, *α*_*s*_ are the volume fraction of gas phase and sand phase, respectively.

Gas phase mass conservation equation is shown in Equation (2).

(2) $$\frac{\partial }{\partial t}\left(\alpha_{g}\rho_{g}\right)+\nabla \left(\alpha_{g}\rho_{g}v_{g}\right)=0\text{.}$$

where *t* (s) is the time; *ρ*_*g*_ (kg/m^3^) is the density of gas phase; *ν*_*g*_ (m/s) is the velocity vector of gas phase.

Sand phase mass conservation equation is shown in Equation (3).

(3) $$\frac{\partial }{\partial t}\left(\alpha_{s}\rho_{s}\right)+\nabla \left(\alpha_{s}\rho_{s}v_{s}\right)=0\text{.}$$

where *ρ*_*s*_ (kg/m^3^) is the density of sand phase; *ν*_*s*_ (m/s) is the velocity vector of sand phase.

Gas momentum conservation equation is shown in Equation (4).

(4) $$\frac{\partial }{\partial t}\left(\alpha_{g}\rho_{g}v_{g}\right)+\nabla \left(\alpha_{g}\rho_{g}v_{g}v_{g}\right)=-\alpha_{g}\nabla p+\nabla \tau_{g}+\alpha_{g}\rho_{g}g+K_{sg}\left(v_{s}-v_{g}\right)\text{.}$$

where *τ*_*g*_ (pa) is the surface stress tensor of gas phase; *p* (pa) is the same pressure shared by two phases; *p*_*g*_ (pa) is gas phase pressure; *g* (m/s^2^) is the acceleration of gravity; *K*_*sg*_ is the momentum exchange coefficient of air phase.

Sand momentum conservation equation is shown in Equation (5).

(5) $$\frac{\partial }{\partial t}\left(\alpha_{s}\rho_{s}v_{s}\right)+\nabla \left(\alpha_{s}\rho_{s}v_{s}v_{s}\right)=-\alpha_{s}\nabla p-\nabla p_{s}+\nabla \tau_{s}+\alpha_{s}\rho_{s}g+K_{gs}\left(v_{g}-v_{s}\right)\text{.}$$

where *τ*_*s*_ (pa) is the surface stress tensor of sand phase; *p*_*s*_ (pa) is the sand phase pressure; *K*_*sg*_ = *K*_*gs*_, and they are the momentum exchange coefficient of air phase and sand phase.

Among them,

$$K_{sg}=\frac{3C_{D}\alpha_{s}\alpha_{g}\rho_{g}}{4d_{s}}\left|v_{s}-v_{g}\right|{\alpha_{g}}^{-2.65}\text{.}$$

$$C_{D}=\frac{24}{\alpha_{g}{\text{Re}}_{s}}\left[1+0.15{\left(\alpha_{g}{\text{Re}}_{s}\right)}^{0.687}\right]\text{.}$$

$$Re_{s}=\frac{\rho_{g}d_{s}\left|v_{s}-v_{g}\right|}{\mu_{g}}\text{.}$$

$$\tau_{g}=\alpha_{g}\mu_{g}\left(\nabla v_{g}+\nabla {v_{g}}^{Τ}\right)+\alpha_{g}\left(\lambda_{g}-\frac{2}{3}\mu_{g}\right)\nabla \cdot v_{g}Ι\text{.}$$

$$\tau_{s}=\alpha_{s}\rho_{s}\left(\nabla v_{s}+\nabla {v_{s}}^{Τ}\right)+\alpha_{s}\left(\lambda_{s}-\frac{2}{3}\mu_{s}\right)\nabla \cdot v_{s}Ι\text{.}$$

$$p_{s}=\alpha_{s}\rho_{s}\theta_{s}+2\rho_{s}\left(1+e_{s}\right){\alpha_{s}}^{2}g_{0}\theta_{s.}$$

where *λ*_*g*_ and *μ*_*g*_ (N·s/m^2^) is the volume viscosity and dynamic viscosity of air; *I* is the second order unit tensor; *e*_*s*_ is sand particle collision restitution coefficient; *g*_*0*_ is the radial distribution function; *d*_*s*_ (mm) is sand particle diameter; *θ*_*s*_ (K) is the sand phase temperature.

This research used standard *k-ε* for the turbulence model. It was assumed that the turbulence developed fully, and the influence of the source phase was not considered.

Turbulent kinetic energy *k* equation is shown in Equation (6).

(6) $$\frac{\partial }{\partial t}\left(\alpha_{g}\rho_{g}k_{g}\right)+\frac{\partial }{\partial x_{i}}\left(\alpha_{g}\rho_{g}k_{g}U_{gj}\right)=\frac{\partial }{\partial x_{j}}\left[\left(\mu +\frac{\mu_{t}}{\sigma_{k}}\right)\frac{\partial k_{g}}{\partial x_{i}}\right]+G_{kg}+G_{b}-\alpha_{g}\rho_{g}\varepsilon_{g}-Y_{m}\text{.}$$

where *k*_*g*_ (J/kg), *μ*_*t*_ (Pa·s), *ε*_*g*_ (W/kg) represent the turbulent kinetic energy, turbulent viscosity, and turbulent dissipation rate, respectively; *U*_*gj*_ (m/s) is the components of velocity in y directions; *σ*_*k*_ is the Prandtl number for turbulent kinetic energy; *G*_*kg*_ (m^2^/s^3^) represents the generation of turbulent kinetic energy due to the mean velocity gradients; *G*_*b*_ (m^2^/s^3^) is the generation of turbulent kinetic energy due to buoyancy; *Y*_*m*_ (m^2^/s^3^) represents the contribution of the fluctuating dilatation in compressible turbulence to the overall dissipation rate the dissipation ratio of compressible turbulence.

Turbulent dissipation rate ε equation is shown in Equation (7).

(7) $$\frac{\partial }{\partial t}\left(\alpha_{g}\rho_{g}\varepsilon_{g}\right)+\frac{\partial }{\partial x_{i}}\left(\alpha_{g}\rho_{g}\varepsilon_{g}U_{gj}\right)=C_{1\varepsilon }\frac{\varepsilon_{g}}{k_{g}}\left(G_{kg}+C_{3\varepsilon }G_{b}\right)+\frac{\partial }{\partial x_{j}}\left[\left(\mu +\frac{\mu_{t}}{\sigma_{\varepsilon }}\right)\frac{\partial \varepsilon_{g}}{\partial x_{j}}\right]-C_{2\varepsilon }\alpha_{g}\rho_{g}\frac{\varepsilon^{2}g}{k_{g}}\text{.}$$

where *U*_*gi*_ (m/s) is the components of velocity in *x* directions; *σ*_*s*_ is the Prandtl number forturbulent dissipation rate. *C*_*1ε*_, *C*_*2ε*_, *C*_*3ε*_ are constants.

Among them,

$$G_{k}=\mu_{t}\left(\frac{\partial u_{i}}{\partial x_{j}}+\frac{\partial u_{j}}{\partial x_{i}}\right)\frac{\partial u_{i}}{\partial x_{j}}\text{.}$$

$$G_{b}=\beta g_{i}\frac{\mu_{i}}{{\mathrm{Pr}}_{i}}\frac{\partial T}{\partial x_{i}}\text{.}$$

$$\beta =-\frac{1}{\rho }\frac{\partial \rho }{\partial T}\text{.}$$

$$Y_{m}=2\rho \varepsilon {M_{i}}^{2}\text{.}$$

Mi=ka2.

$$a=\sqrt{\gamma RT}\text{.}$$

where *P*_*ri*_ is the turbulent Prandtl number for energy; *g*_*i*_ (m/s^2^) is the component of the gravitational vector in the *i*^th^ direction; *β* (1/K) is the coefficient of thermal expansion; *M*_*i*_ is the turbulent Mach number; *a* (m/s) is the speed of sound.

### 2.2 Geometric modeling and meshing

**Fig. 1:**
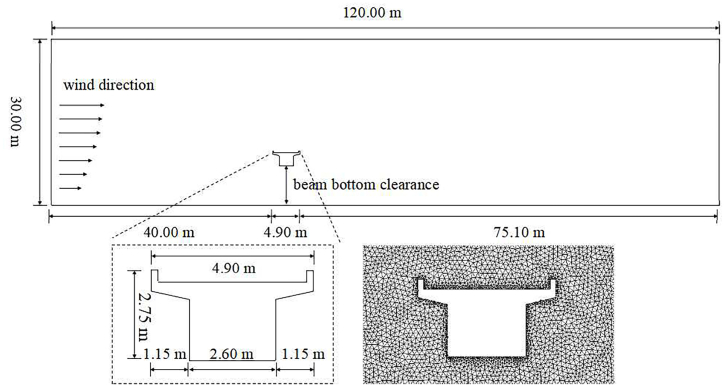
Simulation domain and model mesh.

Sand movement is primarily affected by gravity and drag force, which mainly act in the same plane. ICEM (The Integrated Computer Engineering and Manufacturing code for Computational Fluid Dynamics) was used to build a bridge model, which was simplified into a 2D model to improve the computational efficiency of numerical simulation. Given that the box beam is preferred in wind-sand areas [13,25], the bridge model conforms to the design principle of single-line simply-supported box girder, retaining the beam's external contour while omitting other bridge components. A suspended box beam model was constructed. When the clearance height is 5 m, sand particles still accumulate on the windward side of the beam [25]. Thus, an interval of 1 m was set to build bridge models with clearance heights ranging from 5 m to 15 m. The calculation area was set to 120 m long and 30 m high based on reasonable trial calculations. The bridge model was placed 40 m away from the inlet to allow the wind-sand flow to fully develop. The computational model is shown in Fig. 1. The grid type is hybrid structure to ensure good mesh quality and the accuracy of simulation. The number of grids was about 16 × 10^4^, the average grid quality was 0.90, and the average orthogonal quality and aspect ratio were close to 1, indicating good mesh quality.

### 2.3 Definition of boundary condition

The left side of the model was defined as velocity-inlet. The right side was defined as outflow. The top was set as symmetry, that is, the gradients of velocity and pressure in the direction of the boundary normal were zero. The bridge surface and the ground were defined as wall.

In the simulation experiment, a single vertical wind direction acted on the beam body, and the inflow wind speed was defined by using a user-defined function. The wind-sand speed profiles were defined as shown in Equation (8).

(8) $$V=V_{1}{\left(\frac{z}{z_{1}}\right)}^{0.2}\text{.}$$

where *z* (m) is the height; *z*_*1*_ is the 2 m height; *V*_*1*_ (m/s) is the wind speed at the height of *z*_*1*_; *V* (m/s) is the wind speed at the height of *z*. The inflow wind speeds were set as 10, 15, 20, and 25 m/s.

### 2.4 Parameters

The Euler-Euler two-phase model was used for simulating windblown sand movement in the unsteady numerical analysis. In the simulation, turbulence was assumed to be fully developed, the standard *k-ε* turbulence model was employed, and the standard wall function was used to ensure the calculation accuracy. The Phase coupled SIMPLE method was used for solving, and the iterative residual decreased by 0.001 as convergence. The sand phase volume fraction was set at 0.04. The sand diameter in wind-sand flow ranges from 0.075 to 0.250 mm, but it was set to 0.1 mm for the experiment. The parameters in Fluent were set as follows: sand particles size *d*_*s*_ = 10^−4^ m, sand density *ρ*_*s*_ = 2650 kg/m^3^, sand viscosity *μ*_*s*_ = 0.047 kg/(m·s) [29], air density *ρ*_*g*_ = 1.225 kg/m^3^, and air viscosity *μ*_*g*_ = 1.789 × 10^−5^ Pa·s. The gas pressure was set to standard atmospheric pressure. Schaeffer was used for frictional viscosity, with a packing limiting of 0.63. Considering the force between the gas phase and the solid phase, the lift coefficient was Saffman-mei, and the drag coefficient was the Syamlal-obrien.

### 2.5 Numerical validation

**Fig. 2:**
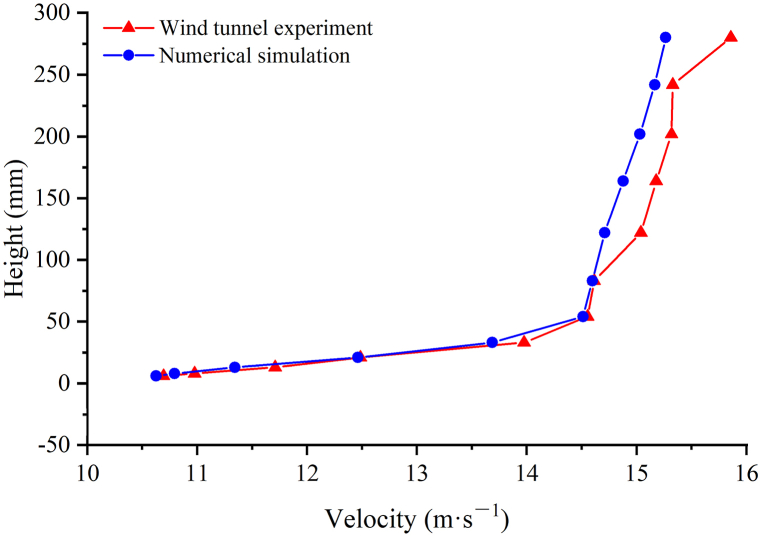
Wind speed profile near-surface from the wind tunnel experiment and numerical simulation.

A wind tunnel experiment was carried out in the Key Laboratory of Desert and Desertification, Chinese Academy of Sciences (CAS) to simulate the characteristics of the wind-sand flow movement around a bridge and validate the numerical simulation results. The wind tunnel test section is 6 m in length and 0.63 m × 0.63 m cross-section, and the boundary layer thickness is 12–15 cm. The bridge model of the wind tunnel experiment was made in the proportion of 1:100, and covered with 36-mesh sandpapers to simulate the surface roughness of the real field bridge. The inflow wind speed was 15 m/s. The numerical simulation used the same computational domain and initial conditions as the wind tunnel experiment. The wind speed profile near the inlet obtained from the wind tunnel experiment and the numerical simulation matched well (Fig. 2), which verified the rationality of the flow field setting in the numerical simulation. The same experiment setting would be used for the flow field calculations of bridges with varying clearance heights.

## 3 Results

### 3.1 Influence of wind speed and clearance height on flow field

**Fig. 3:**
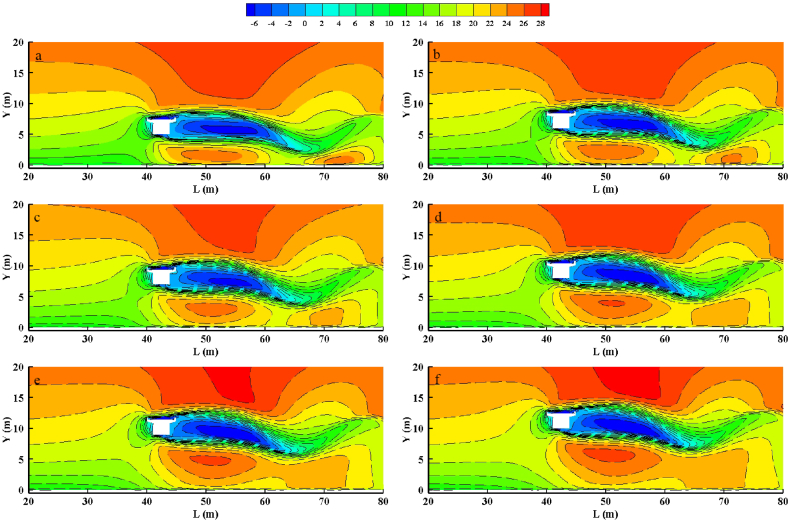
Characteristics of the velocity flow field at different clearances heights. The wind speed is 15 m/s. (a), 5 m; (b), 6 m; (c), 7 m; (d), 8 m; (e), 9 m; (f), 10 m.

Fig. 3 illustrates the air velocity characteristics of bridges with different clearance heights at an inflow wind speed of 15 m/s. The height is represented by Y, and the distance from the entrance is represented by X. The figure shows that when airflow interacts with the bridge, the windward side airflow can be divided into two areas: a deceleration area near the bridge and an acceleration area on the platform shoulder. As the air passes through the bridge, the airflow above it continues to accelerate, forming a strong wind zone. Meanwhile, the airflow beneath the bridge accelerates, creating a high-speed area. On the leeward side, the bridge obstructs the airflow, causing reverse airflow and creating a deceleration area or turbulent flow area. The results indicate that as clearance height increases, the strong wind area above the bridge enlarges. Furthermore, both the height and length of the high-speed zone beneath the bridge increase, with the multiple acceleration zones merging into a single zone. However, the size of the turbulence area on the leeward side remains unchanged as the clearance height increases.

**Fig. 4:**
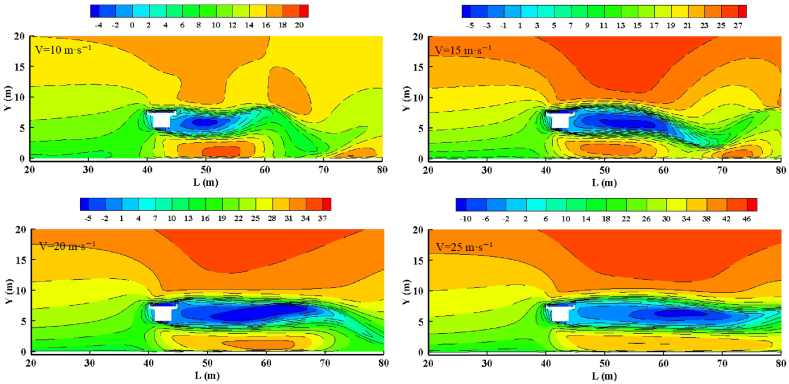
Characteristics of the velocity flow field with different wind speeds. The clearance height is 5 m. (a), 10 m/s; (b), 15 m/s; (c), 20 m/s; (d), 25 m/s.

Fig. 4 shows the airflow velocity field for different wind speeds at a clearance height of 5 m. As the inflow wind speed increases, the changes in airflow on the windward side remain consistent. On the leeward side, however, the upper airflow changes gradually, and the strong wind area shifts toward the bridge. In the turbulence zone on the leeward side, the eddy enlarges as the wind speed increases, with eddy lengths of approximately 15, 20, 25, and 35 m. The range of the wind speed acceleration zone under the bridge also expands. At wind speeds of 20 and 25 m/s, the eddies between 40 and 80 m merge into a continuous region. In summary, the overall position of the airflow field remains unchanged with variations in inflow wind speed.

### 3.2 Influence of clearance height on wind speed

**Fig. 5:**
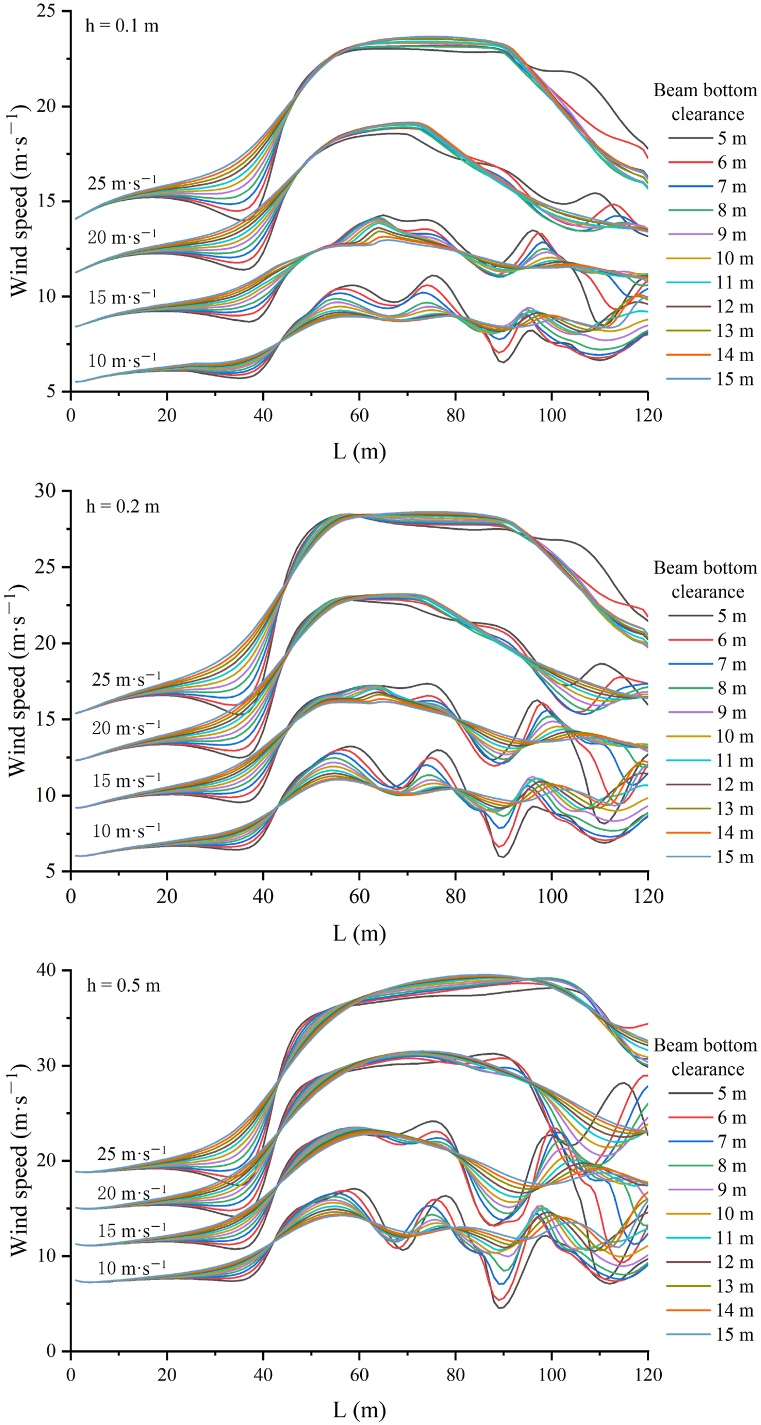
Wind speed curve with different clearance heights under different wind speeds. (a), 0.1 m; (b), 0.2 m; (c), 0.5 m.

The wind-sand flow predominantly moves near the surface, with sand particles concentrated below 0.2 m in heights. To investigate the effect of clearance height on the wind field under the bridge, wind speeds were measured at heights of 0.1 m, 0.2 m, and 0.5 m. Fig. 5 illustrates the wind speed variations at these heights for four different inflow wind speeds. On the windward side of the beam, significant differences in wind speeds were observed for bridges with varying clearance heights. Specifically, as clearance height decreased, wind speed enhancement under the beam increased. Additionally, the variation in wind speed between bridges with different clearance heights became more pronounced as inflow wind speed increased. On the leeward side of the beam, the impact of clearance height on wind speed was even more noticeable. As the inflow wind speed rose, the distance at which wind speed began to decrease significantly also increased. For an inflow wind speed of 10 m/s, wind speed was initially reduced within 10–25 m from the beam. At 15 m/s, this range extended to 30–45 m. For wind speeds of 20 m/s and 25 m/s, wind speed reductions were observed at 30 m and 45 m from the beam, respectively. Furthermore, with increasing inflow wind speed or clearance height, the fluctuation in wind speed between different bridge clearance heights became smaller, and the fluctuation time shortened. In conclusion, regardless of the wind speed, the lower the measuring height, the greater the difference in wind speed fluctuations on the windward side between bridges with different clearance heights. On the leeward side, however, the difference in wind speed fluctuations was smaller. A comparison of the leeward side of the bridge showed that the clearance height had a significant impact on the wind speed on the windward side.

The studies indicate that as wind-sand flow transitions from high-speed area to velocity recovery area, both wind speed and sand transport capacity decrease, leading to sand accumulation on the leeward side of bridge. However, on the windward side, the wind speed is influenced by clearance height and does not decrease once the clearance reaches a certain height. To reduce sand accumulation on both sides of the bridge, it is important to understand how clearance height affects wind speed near the surface on the windward side. The distance over which wind speed decreases and then increases on the windward side was measured to define the deceleration zone. Table 1 shows the deceleration range on the windward side of the bridge at height of 0.2 m, under wind speeds of 10, 15, 20, and 25 m/s. The results show that, for constant wind speeds, higher bridge clearance results in a smaller deceleration area. This is because an increased clearance height enlarges the cross-sectional area for wind-sand flow, leading to smoother velocity fluctuations at the same wind speed. Conversely, for a constant clearance height, the range of the deceleration zone is affected by wind speed. At wind speeds of 10 and 15 m/s, no deceleration zone was observed under the bridge when the clearances exceeded 8 m. For wind speed of 20 and 25 m/s, this threshold increased to above 9 m. Regardless of wind speed, wind speed near the surface did not decrease where the bridge clearance height reached 9 m. This may be due to stronger momentum and turbulence in the airflow at higher wind speeds, which results in a larger range of wind speed fluctuations. In contrast, lower wind speeds cause smaller fluctuations and deceleration zones.

**Table 1:** The range of the deceleration zone on the windward side of the bridge for different clearance heights.

Wind speed (m/s)	**Range of deceleration zone on the windward side (m)**				
	5 m clearance height	6 m clearance height	7 m clearance height	8 m clearance height	9 m clearance height
10	12	9	6	0	0
15	15	12	9	0	0
20	16	15	11	2	0
25	17	15	12	7	0

### 3.3 Definition of the minimum clearance height

The investigations revealed that both the accumulation of sand around the beam and the extent of the deceleration zone on the windward side were highly sensitive to the clearance height. As the clearance height and flow section area increased, the disruption caused by the beam to the surrounding flow field diminished. Once the clearance height reached a certain threshold, the presence of the bridge no longer significantly impacted the ground wind-sand flow. Even with further increases in clearance height, no substantial change in the near-surface wind speed variations under the bridge beams was observed, as the flow was no longer influenced by the beam. Additionally, the wind speeds on the windward side of the beam did not decrease significantly at this clearance height. The wind speed correlation provides insights into the relationship between wind speeds at different clearance heights. If wind speed correlation coefficient for a bridge at a given height aligns closely with that of bridges at higher clearance heights. It indicates a strong correlation with minimal variation. This suggests that the impact of the bridge's clearance height and above on wind speed can be considered negligible. This analysis helps in evaluating the practical implications of varying clearance heights on bridge wind speeds.

To assess the impact of bridge clearance on wind speed patterns, a Spearman correlation analysis was conducted. This analysis evaluated whether each bridge influenced the sand-carrying flow near the surface. Using a wind speed of 20 m/s as an example, Table 2 indicates a strong correlation between near-surface wind speeds under bridges of varying heights. The correlations between adjacent clearance heights were stronger than those between non-adjacent heights. Notably, at three measuring heights, the wind speed correlation coefficient for clearance heights between 10 and 15 m was 1, indicating complete correlation of wind speed fluctuations. For bridges with a clearance height of 9 m or greater, the wind speed correlation coefficients remained consistent, suggesting similar trends in wind speed variations across these bridge heights.

**Table 2:** The correlation coefficient of wind speed under bridges.

h = 0.1 m	Height (m)	5	6	7	8	9	10	11	12	13	14	15
	5	1.000										
	6	0.992	1.000									
	7	0.968	0.989	1.000								
	8	0.940	0.963	0.986	1.000							
	9	0.918	0.938	0.961	0.983	1.000						
	10	0.915	0.935	0.959	0.980	0.998	1.000					
	11	0.915	0.935	0.959	0.980	0.998	1.000	1.000				
	12	0.915	0.935	0.959	0.980	0.998	1.000	1.000	1.000			
	13	0.915	0.935	0.959	0.980	0.998	1.000	1.000	1.000	1.000		
	14	0.915	0.935	0.959	0.980	0.998	1.000	1.000	1.000	1.000	1	
	15	0.915	0.935	0.959	0.980	0.998	1.000	1.000	1.000	1.000	1.000	1
h = 0.2 m	Height (m)	5	6	7	8	9	10	11	12	13	14	15
	5	1.000										
	6	0.981	1.000									
	7	0.952	0.983	1.000								
	8	0.914	0.945	0.972	1.000							
	9	0.897	0.933	0.957	0.987	1.000						
	10	0.897	0.933	0.957	0.987	1.000	1.000					
	11	0.897	0.933	0.957	0.987	1.000	1.000	1.000				
	12	0.897	0.933	0.957	0.987	1.000	1.000	1.000	1.000			
	13	0.897	0.933	0.957	0.987	1.000	1.000	1.000	1.000	1.000		
	14	0.897	0.933	0.957	0.987	1.000	1.000	1.000	1.000	1.000	1.000	
	15	0.897	0.933	0.957	0.987	1.000	1.000	1.000	1.000	1.000	1.000	1.000
h = 0.5 m	Height (m)	5	6	7	8	9	10	11	12	13	14	15
	5	1.000										
	6	0.990	1.000									
	7	0.961	0.983	1.000								
	8	0.940	0.959	0.983	1.000							
	9	0.939	0.959	0.982	0.999	1.000						
	10	0.939	0.959	0.982	0.999	1.000	1.000					
	11	0.939	0.959	0.982	0.999	1.000	1.000	1.000				
	12	0.939	0.959	0.982	0.999	1.000	1.000	1.000	1.000			
	13	0.939	0.959	0.982	0.999	1.000	1.000	1.000	1.000	1.000		
	14	0.939	0.959	0.982	0.999	1.000	1.000	1.000	1.000	1.000	1.000	
	15	0.939	0.959	0.982	0.999	1.000	1.000	1.000	1.000	1.000	1.000	1.000

## 4 Discussion

When airflow moves near a beam, it is obstructed, creating distinct velocity zones: a high-speed area, a concentration acceleration area, a deceleration area, and a low-speed turbulent area. These zones are formed above and below the bridge, as well as on the windward and leeward sides of the bridge. As wind speed increases, the ranges of concentrated acceleration zone and turbulent zone expand, indicating that wind speed has a significant impact on airflow under the bridge. This finding aligns with other studies on the flow field around the bridge [30]. Increasing clearance height and cross-section area increases the likelihood of sand particles being transported to the leeward side of the beam. On the leeward side, wind velocity attenuation extends further from the beam compared to the windward side, where it attenuates more quickly. When wind speed decreases, the sand transport capacity weakens, causing sand accumulation. Consequently, sand accumulation on the windward side poses a greater risk to railway safety [25]. Both wind speed and bridge clearance height influence the wind flow field under the beam. Increasing clearance height or reducing wind speed helps minimize the beam's disturbance to near-surface airflow [11]. Higher clearances allow wind to pass more uniformly, reducing wind speed attenuation and vortex formation. This results in a more stable wind flow around the bridge and reduced pressure losses due to structural disturbances.

The correlation between wind speed and other variables changes at different measurement heights. Under different wind speeds, the wind speed attenuation range on the windward side also differs. A height below 0.2m is ideal for studying sand particle distribution in the wind-sand flow, providing more accurate measurement. At 0.1m, wind speed is strongly influenced by surface roughness and sand deposition. At 0.2m, the impact of sand disturbance factors is reduced, but at 0.5m, local eddy currents may still impact measurements. Thus, choosing the measurement height based on the wind-sand dynamics and analyzing wind speed correlation coefficients at higher wind speeds is essential to determine the minimum bridge height.

Moreover, wind-sand dynamics are influenced by factors such as sand particle size, underlying surface, vegetation coverage and the wind-sand environment, all of which exhibit spatial and temporal variations [31]. The study's simplified model and single environmental condition limit its applicability. Taking sand particle size as an example, Table 3 shows the deceleration zone on the windward side of the bridge for different sand particle diameter. It shows that smaller sand particle size results in a larger deceleration area on the windward side. For instance, when the sand particle size is 0.12 mm, wind speed does not decrease at a clearance height of 8 m. The attenuation of wind speed on the windward side changes with different sand particle sizes. Larger sand particles settle faster, reducing their impact on wind speed attenuation. As a result, they cause less eddying and separation in the wind flow, resulting in less overall attenuation. The minimum bridge clearance is not a fixed value. It should consider local wind and surface conditions in the engineering design. Sand accumulation under bridges should be studied with particular attention to specialized wind-sand environments, such as those found in alpine regions or flowing sand dunes. For bridge in alpine environments, factors such as sand particle size, sand-moving wind and temperature should be considered. In flowing sand environments, attention must be paid to surface sand cover, sand transport and other environmental factors [32]. Furthermore, the structural integrity of railway bridges including bridge piers and the bridge-road transition section (side piers) affects wind-sand flow below the bridge and influences the determination of the minimum clearance height [33].

**Table 3:** Range of the deceleration zone on the windward side of the bridge for different sand particle diameters.

Sand particle diameter (mm)	**Range of deceleration zone on the windward side (m)**				
	6 m clearance height	7 m clearance height	8 m clearance height	9 m clearance height	10 m clearance height
0.08	15	11	5	0	0
0.10	15	11	2	0	0
0.12	14	9	0	0	0

In addition to determining the minimum clearance height, the shape and structure of bridges in windblown sand areas should be improved to be more streamlined, minimizing wind-sand flow interference. Wind-sand flow guiding devices can be installed around the bridge to change the sand flow direction and minimize its impact on the windward side. For existing bridges, regular inspections or sand clearing activities should be conducted on the windward side, in line with prevailing wind directions. If the wind-sand hazards are serious, sand-guiding measures should be implemented on the leeward side where sand deposition occurs.

## 5 Conclusions

This study investigated the variation characteristics of windblown sand flow under the railway bridge beams with clearance heights ranging from 5 to 15 m at different inflow wind speeds, using CFD numerical simulation. The following conclusions are drawn.

(1) Regardless of wind speed or clearance height, the locations of each wind speed zone remained consistent. These zones can be divided into the deceleration zone, acceleration zone, high-speed zone and turbulence zone. As the clearance height increased, the ranges of the high-speed and acceleration zones under the bridge expanded, while the turbulence zone on the leeward side remained unchanged.
(2) At a certain bridge clearance height, wind speed on the windward side no longer decreased further as the clearance height increased. The clearance height directly influenced sand deposition on the windward side of the beam. On the leeward side of the bridge, as wind speed increased, the position of wind speed deceleration moved further from the bridge.
(3) When the bridge clearance height reached 9 m, wind speeds on the windward side of the beam at various measuring heights were no longer affected by the beam, preventing sand particles deposition. The correlation coefficients of wind speeds under bridges with clearance heights of 9 m and above were equal, indicating consistent trends in near-surface wind-sand movement. Therefore, the minimum clearance height in windblown sand areas should be 9 m.

## CRediT authorship contribution statement

**Xian Zhang:** Writing – original draft. **Shengbo Xie:** Writing – review & editing, Funding acquisition. **Yingjun Pang:** Writing – review & editing.

## Data availability statement

The data presented in this study are available within the article.

## Funding

This research project was supported by the 10.13039/501100001809National Natural Science Foundation of China (grant nos. 42077448 and 42477505), the Western Young Scholars project of the Chinese Academy of Sciences of China (grant no. xbzglzb2022024), the Natural Science Foundation of Gansu Province for Distinguished Young Scholars (grant no. 22JR5RA049), and the Ordos Science and Technology Plan (grant no. 2021EEDSCXQDFZ013).

## Declaration of competing interest

The authors declare that they have no known competing financial interests or personal relationships that could have appeared to influence the work reported in this paper.
